# A Complex Interaction between *Rickettsia conorii* and Dickkopf-1 – Potential Role in Immune Evasion Mechanisms in Endothelial Cells

**DOI:** 10.1371/journal.pone.0043638

**Published:** 2012-09-18

**Authors:** Elisabeth Astrup, Tove Lekva, Giovanni Davì, Kari Otterdal, Francesca Santilli, Erik Øie, Bente Halvorsen, Jan Kristian Damås, Didier Raoult, Giustina Vitale, Juan P. Olano, Thor Ueland, Pål Aukrust

**Affiliations:** 1 Institute of Clinical Medicine, Akershus University Hospital, Lørenskog, Norway; 2 Research Institute of Internal Medicine, Oslo University Hospital Rikshospitalet, Oslo, Norway; 3 Section of Specialized Endocrinology, Oslo University Hospital Rikshospitalet, Oslo, Norway; 4 Section of Clinical Immunology and Infectious Diseases, Oslo University Hospital Rikshospitalet, Oslo, Norway; 5 Faculty of Medicine, University of Oslo, Oslo, Norway; 6 Department of Cancer Research and Molecular Medicine, Norwegian University of Science and Technology, Trondheim, Norway; 7 Department of Infectious Diseases, St. Olavs Hospital, Trondheim, Norway; 8 Center of Excellence on Aging, University of Chieti, Palermo, Italy; 9 Department of Medicine, School of Medicine, Palermo, Italy; 10 Unité des Rickettsies, Faculté de Médecine, Université de la Mediterranée, Marseille, France; 11 Department of Pathology, University of Texas Medical Branch, Galveston, Texas, United States of America; University of Minnesota, United States of America

## Abstract

The pathophysiological hallmark of spotted fever group rickettsioses comprises vascular inflammation. Based on the emerging importance of the wingless (Wnt) pathways in inflammation and vascular biology, we hypothesized that Dickkopf-1 (DKK-1), as a major modulator of Wnt signaling, could be involved in the pathogenesis in rickettsial infections. Our major findings were: (i) While baseline concentration of DKK-1 in patients with *R. conorii* infection (n = 32) were not different from levels in controls (n = 24), DKK-1 rose significantly from presentation to first follow-up sample (median 7 days after baseline). (ii) *In vitro* experiments in human umbilical vein endothelial cells (HUVECs) showed that while heat-inactivated *R. conorii* enhanced the release of interleukin-6 (IL-6) and IL-8, it down-regulated the release of endothelial-derived DKK-1 in a time- and dose-dependent manner. (iii) Silencing of DKK-1 attenuated the release of IL-6, IL-8 and growth-related oncogene (GRO)α in *R. conorii*-exposed HUVECs, suggesting inflammatory effects of DKK-1. (iv) Silencing of DKK-1 attenuated the expression of tissue factor and enhanced the expression of thrombomodulin in *R. conorii*-exposed HUVECs suggesting pro-thrombotic effects of DKK-1. The capacity of *R. conorii* to down-regulate endothelial-derived DKK-1 and the ability of silencing DKK-1 to attenuate *R. conorii*-induced inflammation in endothelial cells could potentially reflect a novel mechanism by which *R. conorii* escapes the immune response at the site of infection.

## Introduction

Rickettsiae are Gram-negative intracellular bacteria transmitted by arthropod vectors. Rickettsioses can present clinically in an array of different clinical symptoms; the most consistent being fever, myalgia, lymphadenopathy, and headache, with or without eschar and/or maculopapular eruption [1]. The clinical spectrum of spotted fever group (SFG) rickettsioses varies in severity from mild to potentially lethal disease with systemic multi-organ involvement such as in some cases of Rocky Mountain spotted fever (RMSF) caused by *Rickettsia rickettsii* (*R. rickettsii*) and Mediterranean spotted fever (MSF) caused by *R. conorii* [1].

The pathophysiological hallmark of SFG rickettsioses comprises infection of endothelial cells and subsequent perivascular infiltration of T cells and monocytes/macrophages, resulting in vasculitis, with increased microvascular permeability and in some cases, cerebral and pulmonary edema [1], [2]. This interaction between microbe and endothelial cells triggers innate immune responses, including the production of several cytokines by endothelial and non-endothelial cells, representing both beneficial (i.e., anti-microbial) and detrimental (e.g., tissue destruction and excessive inflammation) responses in relation to the infected host [1], [2].

Immune escape or immune evasion is an important mechanism for microbe survival within the host to avoid innate and adaptive immune responses. Such mechanisms are of importance in viral, bacterial and parasitic infection, and are thought to be of particular relevance for intracellular bacterial infection [3]. The immune evasion mechanisms for bacteria involve molecular mimicry, suppression of antibodies, hiding inside cells and inhibition of phagocytosis [3]. There are also some reports suggesting that such mechanisms could be operating in Rickettsial infection [2], [4], but these issues are far from clear.

The wingless (Wnt) pathway involves a large number of proteins that participate in a range of developmental and physiological processes including cardiac and vascular development. Wnt signaling is regulated by multiple families of secreted antagonists such as soluble frizzled related receptors and dickkopfs (DKKs). The best studied of these is DKK-1, which dampens the Wnt signal by binding to the LPR5/6 receptor and a cell surface co-receptor, Kremen-1/2, promoting internalization of the receptor complex [5]. In adults, DKK-1 has been implicated in the pathogenesis of bone disease, cancer, Alzheimer's disease, and brain ischemia [5], [6]. Recent studies also point to an important role of the Wnt signaling pathways and DKK-1 in the regulation of inflammation. Thus, activation of the canonical Wnt/β-catenin pathway induces proliferation and survival of endothelial cells, enhances monocyte adhesion, and regulates transendothelial migration of monocytes [7]–[10]. Also, the destructive effect of tumor necrosis factor α (TNFα) on joints in rheumatoid arthritis was found to involve DKK-1 [6], and we have shown that platelet- and endothelial-derived DKK-1 could contribute to vascular inflammation in atherosclerosis [11]. The Wnt signaling pathway has recently also been implicated in the pathogenesis of certain infectious disorders including septicemia [12] and infection by intracellular pathogens (i.e., Chlamydia infection) [13].

Based on the emerging importance of the Wnt signaling pathways in inflammation and vascular biology, we hypothesized that DKK-1, as a major modulator of Wnt signaling, could be involved in the pathogenesis of rickettsial infections. Here, this hypothesis was investigated by various experimental approaches including *in vivo* studies in patients with *R. conorii* infection as well as *in vitro* studies focusing on the role of DKK-1 in the interaction between *R. conorii* and endothelial cells using heat-inactivated *R. conorii* as a model for the early phase of this interaction.

## Methods

### Patients and controls

Thirty-two consecutively recruited patients (17 women and 15 men, 19–90 [mean 61.5] years of age) with MSF, confirmed by seroconversion, admitted to the Termini Imerese Hospital Palermo, Palermo, Italy, between June and September 2005, were included in the study [14]. They all had characteristic signs and symptoms of active MSF (fever, eschar at the site of tick bite, and maculopapular rash). The duration of illness before diagnosis was less than 2 weeks. One patient received cephalosporin, two ciprofloxacin, one received no treatment, whereas the remaining patients were treated with tetracycline (500 mg 4 times a day for 7 days). All patients with MSF had seroconversion with increases in the levels of anti-*R. conorii* antibodies as assessed by enzyme-linked immunosorbent assay (ELISA) and indirect immunofluorescence assay [15]. Twenty-four healthy subjects (9 women and 15 men, aged 21–67 [mean 43.4] years), recruited from the same area of Italy, were included in the study as controls. All controls were healthy individuals as assessed by disease history, clinical examination and normal C-reactive protein levels, with no signs of concomitant disease that could interfere with DKK-1 levels. All patients were invited to sign the medical records and received information and consented that their blood sampling might be used for future investigations regarding their disease.

All parts of the study were approved by the local ethical committee and conducted according to the ethical guidelines from the declaration of Helsinki (Ref IRB, Termini Imerese Hospital Palermo, Palermo, Italy and Regional Committee for Medical and Research Ethics, South-East, Norway, ref 248-08/239 2008/230).

### Blood sampling protocol

Blood was collected both at first presentation (less than 2 weeks after the onset of the symptoms and before specific treatment), and at two times during follow-up (median 7 days and >21 days after baseline samples). At the last blood sampling, all patients had recovered and were free of clinical symptoms. Peripheral venous blood was drawn into pyrogen-free, vacuum blood collection tubes without any additives, immediately immersed in melting ice and allowed to clot before centrifugation at 2000*g* for 10 minutes. Serum was stored at −80°C until analysis and samples were thawed less than three times.

### Bacteria

*R. conorii* (Malish strain) were grown in Vero cell monolayers in 150 cm^2^ tissue culture flasks, cultured in Modified Eagle Medium (MEM; Gibco, Paisley, UK), supplemented with 4% fetal calf serum (FCS) and 2 mM L-glutamine. Heavily infected cells (5 days post-inoculation) were harvested with sterile glass beads and pelleted by centrifugation at 10,000*g* for 15 minutes. Antigens used were purified by sucrose gradients and were entire bacterial antigens. The pellets were resuspended in sterile distilled water so that each suspension had the same density of organisms as determined microscopically at ×100 magnification [16]. Different dilutions of this suspension were used for *in vitro* experiments. The batch contained 10 million rickettsiae/ml and was diluted in phosphate buffered saline (PBS) before being added to the cell cultures. Heat-inactivated organisms were obtained by heating at 60°C for 30 minutes. All experiments with live bacteria were conducted under Biosafety Level 3 (BSL3) conditions.

### Endothelial cell culture

Human umbilical vein endothelial cells (HUVECs) were obtained from umbilical cord veins by digestion with 0.1% collagenase A (Roche Diagnostics GmbH, Mannheim, Germany) and cultured as previously described [17]. HUVECs were passaged by treatment with 0.05% trypsin-EDTA (Gibco) and grown to confluence for 3 to 5 days. The HUVECs were used at passage levels 4–9. The medium was then discarded, and HUVECs were stimulated with different concentrations of heat-inactivated *R. conorii* in MCDB-131 serumfree medium (Sigma St. Louis MO), except for the long-term stimulations (up to 120 hrs), when it was supplemented with 50% fetal bovine serum (Gibco). In a separate set of experiments, HUVECs were transfected with small interfering RNA (siRNA) against DKK-1 prior to exposure to heat-inactivated *R. conorii* (see below). Cell-free supernatants and cell pellets were harvested after various time points and stored at −80°C until analyses. The density of the endothelial cells were ∼86.000 cells per well, and as an example, 4×10^5^ bacteria per ml will give approximately 1.4 bacteria/cell. The toxicity in cell cultures was examined for lactate dehydrogenase leakage using a cytotoxicity detection kit (Roche Applied Science, Indianapolis, IN).

### Cultures of THP-1 macrophages and vascular smooth muscle cells (SMC)

In a separate set of experiments, Tamm-Horsfall protein 1 (THP-1) macrophages and vascular SMC were incubated with heat-inactivated *R. conorii*. The *human monocytic cell line THP-1* (American Type Culture Collection, Rockville, MD) was cultured in RPMI 1640 (PAA laboratories, Pasching, Austria), supplemented with 2.5% fetal bovine serum. Before the experimental start, the THP-1 cells were differentiated into macrophages by incubation for 24 hours with phorbol myristate acetate (PMA, 100 nM; Sigma) before resting for additional 48 hours, and further incubated with or without heat-inactivated *R. conorii*.

*Human aortic SMC* were obtained from PromoCell GmbH (Heidelberg, Germany) and grown in SMC Growth Medium 2 with complete supplement mix (PromoCell). At 90% confluence, the culture was trypsinized and replated. At experimental start, the cells were cultured in Optimem with Glutamax (Gibco-Invitrogen, Carlsbad, CA) with or without heat-inactivated *R. conor*ii. At different time points, cell-free supernatants were harvested and stored at −80°C.

### Preparation and stimulation of platelets

Preparation and stimulation of platelets were performed as previously described [18]. Briefly, one-fourth volume of acid-citrate-dextrose (85 mM trisodium citrate, 71.4 mM citric acid and 111 mM glucose, pH = 4.5) was added to platelet-rich plasma prior to centrifugation at 1,500*g* for 7 minutes at 22°C. The platelets were then resuspended in MCDB-131 media (5×10^8^ platelets/ml) before stimulation with *R. conorii* for 1 hour. DKK-1 levels were determined in platelet-free solution (centrifugation of platelet suspension for 5 minutes at 10,000*g*) at the end of the experiment.

### Whole blood experiments

Human whole blood from four different healthy donors was collected. The blood was anti-coagulated with lepirudin (50 µg/ml), and immediately split into sterile polypropylene tubes (1.8 ml NUNC cryotubes) for incubation. The blood was incubated under tilting for 4 hours at 37°C with and without heat-inactivated *R. conorii* diluted in PBS with CaCl_2_ and MgCl_2_ (Sigma). Further activation was blocked by adding EDTA (10 mM). The tubes were centrifuged for 15 minutes at 4000*g* at 4°C. Plasma was stored at −80°C until being analyzed for DKK-1-release.

### Preparation and transfection of siRNA

siRNAs with the following sense and antisense sequences were used: DKK-1, first strand (sense), 5′-GCUUCACACUUGUCAGAGAtt-3′, second strand (antisense), 5′-UCUCUGACAAGUGUGAAGCct-3′. Scrambled control, a non-targeting siRNA (siSCR), was used as control. All sequences were provided from Applied Biosystems (Foster City, CA). For transfection, 50 nM siRNA duplexes and 6 µl HiPerFect transfection reagent (Qiagen, Hilden, Germany) were prepared in OptiMem with glutamax-1 (Gibco-Invitrogen, Carlsbad, CA), and added when HUVECs reached 70% confluence at a final volume of 300 µl in 12-well plates (Costar, Cambridge, MA). The concentration of siRNA duplexes (50 nM) was based on dose-response efficacy experiments and toxicity studies (lactate dehydrogenase [LDH] release), and importantly, there was no difference in LDH-release between siDKK-1 and siSCR exposed HUVECs. After 6 hours, 300 µl medium with 10% FCS was added to the cells for overnight incubation. After 24 hours incubation, the cells were cultured with or without *R. conorii* as described above. In a separate experiment, the transfected cells were incubated with or without different concentration of recombinant DKK-1 (R&D Systems, Minneapolis, MN).

### Isolation of nuclear and cytoplasmic extract of HUVECs

The cells were seeded in 12 well plates and grown to confluence. They were treated for 15 and 120 min with vehicle and R. conorii, (n = 4), and washed twice with cold PBS. Thereafter cells were scraped and resuspended in cell lysis buffer (10 mM Tris-HCl pH 7.4, 10 mM NaCl, 3 mM MgCl, protease inhibitor cocktail tablet [EDTA-free; Roche, Basel, Switzerland], 1 mM phenylmethylsulfonyl fluoride, 0.3% Igepal) on ice, and centrifuged at 5000*g* for 6 minutes at 4°C. The supernatant fraction (cytoplasmic extract) was removed, centrifuged again at 5000*g* for 6 minutes at 4°C and supernatants were stored at 80°C until further analyses. The cell pellet (nuclear fraction) was washed twice in cell lysis buffer, (10 mM Tris-HCl pH 7.4, 10 mM NaCl, 3 mM MgCl, protease inhibitor cocktail tablet [EDTA-free; Roche, Basel, Switzerland], 1 mM phenylmethylsulfonyl fluoride) on ice, and resuspended in another cell lysis buffer (50 mM Hepes pH 7.5, 125 mM NaCl, 0.5% Igepal, 1 mM EDTA, 5% glycerol, 1 mM NaF, protease inhibitor cocktail tablet [EDTA-free; Roche], 1 mM phenylmethylsulfonyl fluoride), briefly sonicated (approx. 2 sec), centrifuged at 5000*g* for 6 minutes at 4°C and supernatants were stored at 80°C until further analyses.

### Intracellular total β-catenin measurements

Both phosphorylated and unphosporylated β-catenin was measured by ELISA from R&D Systems in nuclear and cytoplasmic cell fractions and correlated to total protein (BCA Protein Assay Kit; Pierce, Rockford, IL).

### Real-time quantitative RT-PCR

Total RNA was extracted from HUVECs using RNeasy columns (Qiagen), subjected to DNase I treatment, and stored in RNA storage solution (Ambion, Austin, TX) at −80°C. Primers for DKK-1 (forward primer [FP]: 5′-GGGAATTACTGCAAAAATGGAATA-3′ and reverse primer [RP]: 5′-ATGACCGGAGACAAACAGAAC-3′), interleukin-6 (IL-6) (FP: 5′-AGCCCTGAGAAAGGAGACATGTA-3′ and RP: 5′-CATCTTTGGAAGGTTCAGGTTGT-3′), monocyte chemoattractant protein 1 (MCP-1) (FP: 5′-AAGCTGTGATCTTCAAGACCATTGT-3′ and RP: 5′-TGGAATCCTGAACCCACTTCTG-3′), growth-related oncogene (FP: 5′-TGCGCCCAAACCGAAG-3′ and RP: 5′-TGCAGGATTGAGGCAAGCTT-3′), IL-8 (FP: 5′-GCCAACACAGAAATTATTGTAAAGCTT-3′ and RP: 5′-CCTCTGCACCCAGTTTTCCTT-3′), plasminogen activator inhibitor (PAI)-1 (FP: 5′-AGGCTGACTTCACGAGTCTTTCA-3′ and RP: 5′-GCTGAGACTATGACAGCTGTGGAT-3′), tissue factor (TF) (FP: 5′-GCGCTTGAGGCACTAAAAT-3′ and RP: 5′-TTTGCTTTTCCAATCTCCTGA-3′), and thrombomodulin (FP: 5′-CCCAACACCCAGGCTAGCT-3′ and RP: 5′-CGTCGATGTCCGTGCAGAT-3′) were designed using the Primer Express software, version 2.0 (Applied Biosystems). Quantification of mRNA was performed using the ABI Prism 7500 (Applied Biosystems). Gene expression of the housekeeping gene β-actin (Applied Biosystems) was used for normalization.

### ELISA

Levels of IL-6, IL-8, MCP-1, GROα, and DKK-1 were measured by ELISAs obtained from R&D Systems. The intra- and inter-assay coefficients of variations were <10% for all ELISAs. To further minimize run-to-run variability, serial samples from a given individual were analyzed on the same tray.

### Statistical methods

*In vivo* data (i.e. serum) were analyzed by non-parametric statistics: Mann-Whitney U test for comparing patients and controls and Wilcoxon signed-rank test for comparing changes in individuals over time. In addition, linear regression was used to verify differences in DKK-1 levels due to differences in age between patients and controls (i.,e., log transformed DKK-1 levels as dependent variable and age and group as independent in a forced linear regression). For *in vitro* data, parametric statistics were used. When two groups were compared (i.e. siRNA experiments comparing the same time point or *R. conorii* concentration between siSCR and siDKK-1), unpaired t-tests were used, while in some dose experiments with 3 or more groups to compare (i.e. un-stimulated and 2 or 3 doses), a one way ANOVA was used first and if significant, unpaired t-test was used to compare differences with the un-stimulated situation. The level of significance was set at p<0.05.

## Results

### Serum levels of DKK-1 in MSF patients

**Figure 1:**
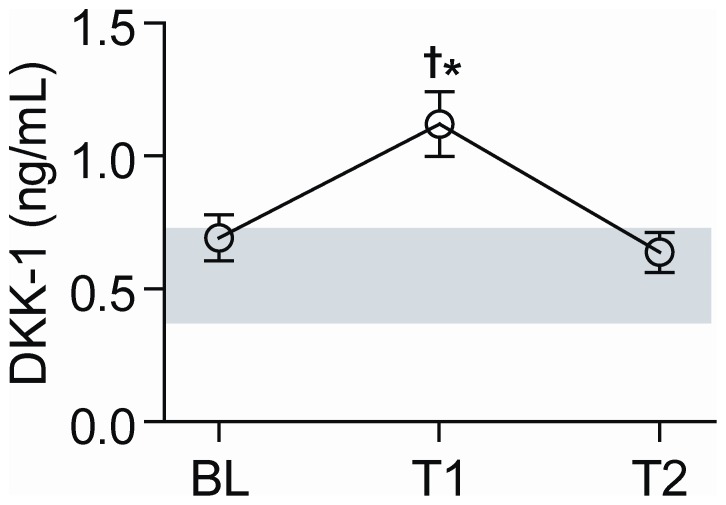
Serum levels of DKK-1 in patients with MSF. The figure shows serum DKK-1-levels measured at baseline (BL), first follow-up (T1, median 7 days after baseline) and after recovery (T2, >21 days after baseline) in 32 patients with MSF and in 24 healthy controls (grey area represent mean±95% CI of healthy controls). Data are given as mean±SEM. †*P*<.001 *vs.* baseline, *P<.001 *vs.* controls.

While baseline (less than 2 weeks after the onset of the symptoms and before specific treatment) concentrations of DKK-1 in patients with MSF (n = 32) were not different from levels in healthy controls (n = 24), DKK-1 rose significantly from presentation to first follow-up sample (median 7 days after baseline), reaching levels that were 2-fold higher than in healthy individuals (Figure 1). This late surge is in contrast to the immediate increase in levels of established inflammatory parameters such as IL-8 and circulating adhesion molecules that are seen during *R. conorii* infection [14]. After recovery (>21 days after baseline), DKK-1 decreased to reach the same levels as on first presentation (Figure 1).

The control group was somewhat younger than the MSF patients (see Methods), but we found no significant or non-significant trend for the association between age and DKK-1 levels in neither patients nor controls (data not shown). However, as there was a significant difference in age between patients and controls, we wanted to make it clear that the differences in DKK-1-levels were not due to age differences and importantly, a significant difference (p = 0.003) in DKK-1 levels at time point 2 (first follow-up sample) between patients and controls was observed also when adjusting for age using linear regression. Blood samples from the control group were only collected at one time point. However, when collecting serum samples longitudinally (baseline, 1 week and 3 weeks) from 9 additional healthy controls to evaluate the stability of DKK-1 levels over time, the coefficient of variation for DKK-1 levels was 16.7±12.6%. Although this reflects some variation, it is clearly below the increase in DKK-1 levels from baseline to time point 2 in MSF patients (Figure 1).

### Effects of heat-inactivated *R. conorii* in endothelial cells

**Figure 2:**
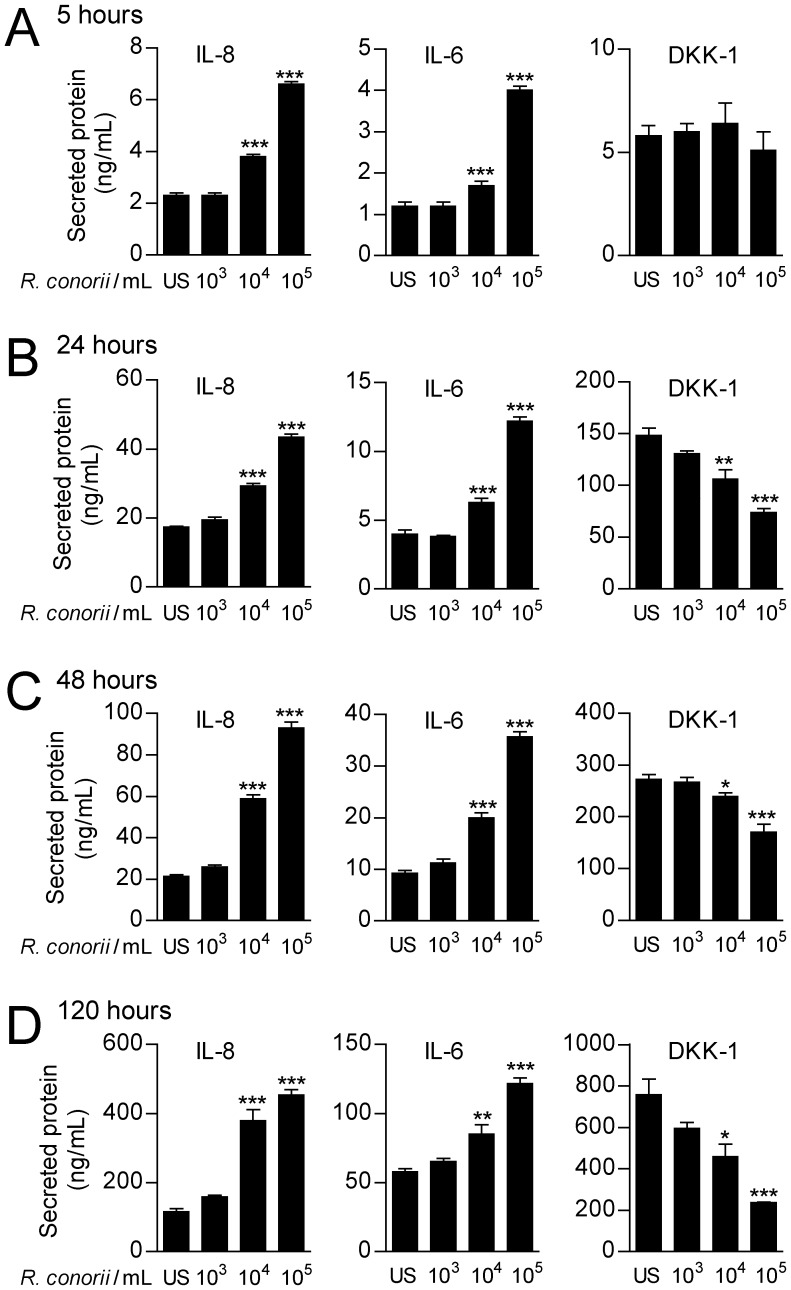
The effect of *R. conorii* on cytokine in release in HUVECs. The figure shows the effect of different concentrations of heat-inactivated *R. conorii* on the release of IL-8 (**left panels**), IL-6 (**middle panels**) and DKK-1 (**right panels**) in HUVECs after culturing for 5 (**A**), 24 (**B**), 48 (**C**) and 120 (**D**) hours. Data are given as mean±SEM (n = 6). *P<.05, **P<.01 and ***P<.001 *vs.* un-stimulated cells (US).

We have previously shown that endothelial cells release large amounts of DKK-1 upon activation [11], and these cells are also clearly relevant in relation to rickettsial infection. We therefore next examined the ability of heat-inactivated *R. conorii* to modulate DKK-1 release in HUVECs. As shown in Figure 2, *R. conorii* promoted a significant decrease in the release of DKK-1 from HUVECs in a dose- and time-dependent manner, with a maximal suppression after 120 hours at a concentration of 10^5^/ml. In contrast to this suppressive effect on DDK-1 release, *R. conorii* enhanced the release of the prototypical inflammatory cytokines IL-8 and IL-6, emphasizing that *R. conorii* may differentially regulate DKK-1 and inflammatory cytokines (i.e., IL-8 and IL-6) in HUVECs (Figure 2). The culture media was not changed during the culture period (120 hours), and the increase in IL-6 and IL-8 levels over time most probably reflects accumulation of these proteins in combination with enhanced synthesis.

### Effects of heat-inactivated *R. conorii* on DKK-1 release in other cells with relevance to MSF

**Figure 3:**
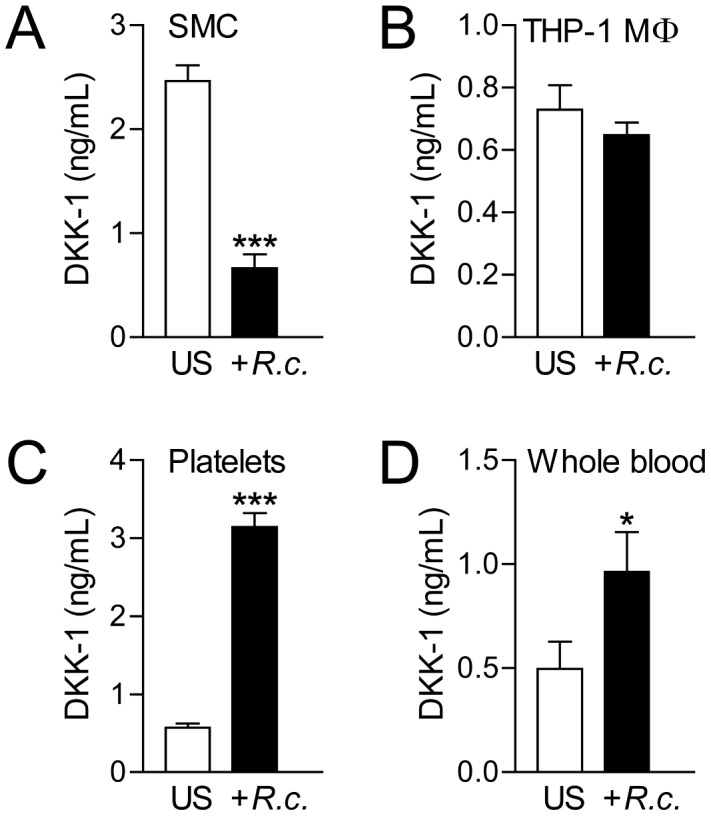
The effect of *R. conorii* on DKK-1 release in various cells and cell lines. The figure shows the effect of heat-inactivated *R. conorii* (10^5^/ml **A**–**C**, 10^4^/ml **D**) on the release of DKK-1 in cell free supernatants in vascular SMC (**A**, culture time 20 hours), THP-1 macrophages (**B**, culture time 20 hours), washed platelets (**C**, culture time 1 hour) and whole blood (**D**, culture time 4 hours). Data are given as mean±SEM (n = 4–6). *P<.05 and ***P<.001 *vs.* un-stimulated cells (US).

Platelets have been shown to release significant amounts of DKK-1 upon activation [11], and we have previously shown that heat-inactivated *R. Africae* could promote platelet action [19]. To examine wether platelets and other cells with relevance to *R. conorii* infection could contribute to serum levels of DKK-1 during MSF, we examined the ability of heat-inactivated *R. conorii* to modulate the release of DKK-1 in vascular SMC, macrophages, whole blood cultures and platelets. As in endothelial cells, *R. conorii* down-regulated DKK-1 release in vascular SMC, but the levels were in general markedly lower than in endothelial cells (Figure 3). In contrast, heat-inactivated *R. conorii* enhanced the release of DKK-1 from washed platelets and whole blood with the same pattern in these two cell cultures, potentially reflecting effects on platelets in the whole blood culture (Figure 3). Finally, *R. conorii* had no effect on the release of DKK-1 in THP-1 macrophages (Figure 3).

### Heat-inactivated *R. conorii* activates the Wnt pathway in endothelial cells

**Figure 4:**
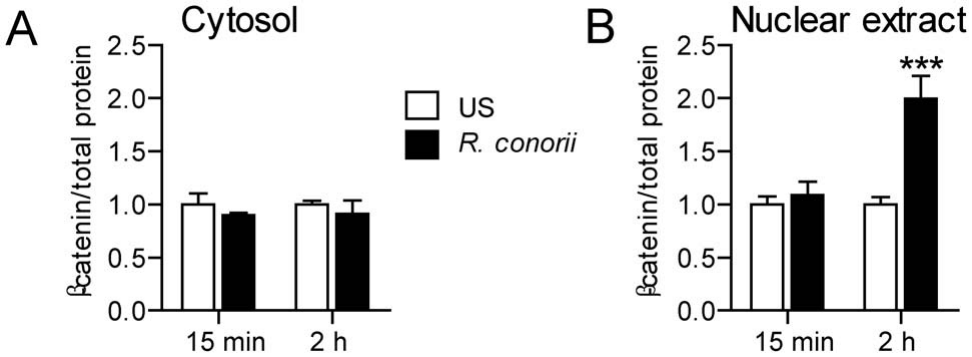
The effect of *R. conorii* on β-catenin activation in HUVECs. The figure shows the effect of heat-inactivated *R. conorii* (400,000/ml) in HUVECs on the accumulation of total (phosphorylated and non-phosphorylated) β-catenin in cytosol (**A**) and within the nucleus (**B**) after culturing for 15 minutes (min) and 2 hours (h). Data are given as mean±SEM (n = 6). ***P<.001 *vs.* un-stimulated cells (US) at the same time point.

To examine if endothelial-derived DKK-1 could modulate the interactions between *R. conorii* and endothelial cells, we first examined if heat-inactivated *R. conorii* could activate the Wnt pathway in these cells. The canonical Wnt/β-catenin pathway stimulates stabilization and accumulation of cytosolic, and then later nuclear β-catenin, which binds to sensitive transcription factors [20]. As shown in Figure 4, heat-inactivated *R. conorii* induced accumulation of β-catenin within the nucleus after 2 hours, suggesting that *R. conorii* promotes activation of canonical Wnt/β-catenin pathway in HUVECs.

### Effect of silencing DKK-1 on the *R. conorii*-mediated induction of inflammatory cytokines in endothelial cells

**Figure 5:**
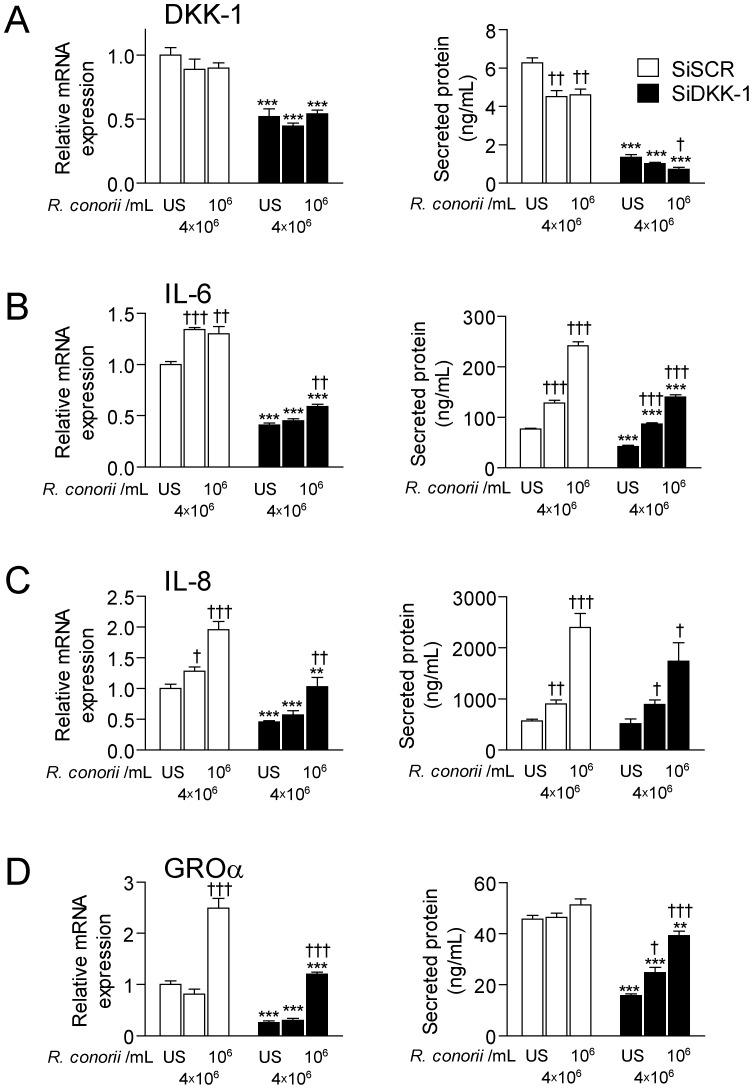
The effect of silencing DKK-1 on the *R. conorii*-induced cytokine levels in HUVECs. The figure shows the effect of different concentrations of *R. conorii* in HUVECs on mRNA expression (left panel) and release of protein in cell supernatants (right panel) of DKK-1 (**A**), IL-6 (**B**), IL-8 (**C**) and GROα (**D**) in the presence (siDKK, filled bars) and absence (siSCR, open bars) of siRNA probes to silence DKK-1. Silencing for 24 hours plus culturing for 6 (mRNA) and 24 (protein) hours respectively. Data are given as mean±SEM (n = 4). **P<.01, ***P<.001 *vs.* siSCR treated cells (control cells). †*P*<.05, ††*P*<.01 and †††*P*<.001 *vs*. un-stimulated (US) siSCR treated cells.

In order to further examine the interaction between *R. conorii* and the Wnt pathway, we transfected HUVECs with siRNA probes to silence DKK-1. HUVEC spontaneously expressed large amounts of DKK-1 at both mRNA and protein levels, and we found successful silencing of DKK-1 as assessed by real-time RT-PCR (∼48%) and by ELISA (∼78%) 48 hours post-transfection (Figure 5). Heat-inactivated *R. conorii* induced a significant increase in IL-6, IL-8 and GROα levels compared with un-stimulated cells, although the increase in GROα was only seen at the mRNA level (Figure 5). Notably, silencing DKK-1 attenuated the *R. conorii*-mediated release of these inflammatory cytokines (Figure 5). This effect was particularly marked for IL-6, but did not reach statistical significance for IL-8 (p = 0,19) (Figure 5). The cells were cultured with heat-inactivated *R. conorii* for 24 hours following the initial 24 hours of transfection. The same patterns were also seen at the mRNA levels (6 hours), suggesting that DKK-1 influences the production and not only the release of these inflammatory mediators (Figure 5). Silencing DKK-1 markedly down-regulated the inflammatory response upon exposure to heat-inactivated *R. conorii*. Still, the *R. conorii*-induced IL-6 and IL-8 response at the protein level, but not the GROα response that was even lower than in un-stimulated cells, was significantly increased as compared to the un-stimulated condition in non-silenced cells (Figure 5). In contrast to the effect on IL-6, IL-8 and GROα, silencing DKK-1 did not modulate the *R. conorii*-induced expression of MCP-1 neither at protein nor mRNA levels (data not shown).

### Effect of silencing DKK-1 on the heat-inactivated *R. conorii*-mediated induction of pro- and anti-thrombotic mediators in endothelial cells

**Figure 6:**
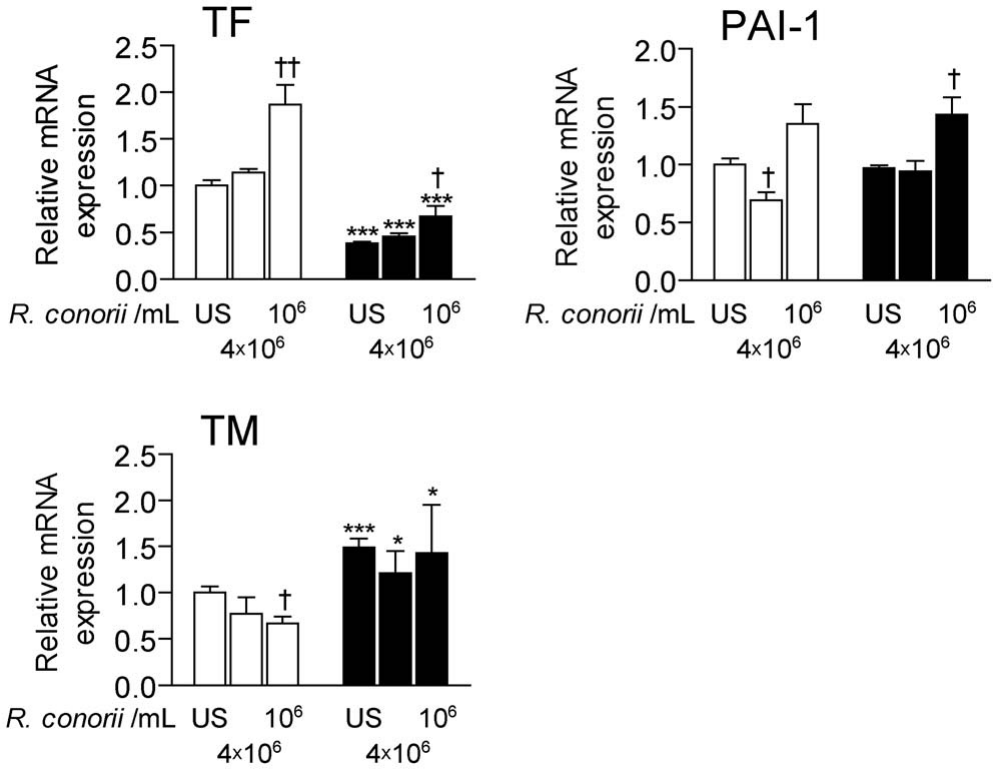
The effect of silencing DKK-1 on the *R. conorii*-induced levels of pro- and anti-thrombotic mediators in HUVECs. The figure shows the effect of different concentrations of *R. conorii* in HUVEC on mRNA expression of tissue factor (TF), plasminogen activator inhibitor (PAI-1) and thrombomodulin (TM) in the presence (siDKK, filled bars) and absence (siSCR, open bars) of siRNA probes to silence DKK-1 after silencing for 24 hours with additional culturing for 6 hours. Data are given as mean±SEM (n = 4). *P<.05, ***P<.001 *vs.* siSCR treated cells (control cells). †*P*<.05 and ††*P*<.01 *vs.* un-stimulated (US) siSCR treated cells.

In addition to vasculitis, thrombus formation related to endothelial cell activation is an important clinical manifestation of SFG rickettsioses [4], [19]. We therefore next examined the effect of silencing DKK-1 on the *R. conorii*-induced expression of endothelial-derived pro- (i.e., TF and PAI-1) and anti- (i.e., thrombomodulin) thrombotic mediators. While heat-inactivated *R. conorii* enhanced the expression of TF in scrambled control cells, silencing DKK-1 attenuated mRNA levels of TF after culturing for 6 hours in both un-stimulated and *R. conorii*-exposed cells (Figure 6). In contrast, whereas *R. conorii* decreased the expression of thrombomodulin in control cells, silencing DKK-1 enhanced mRNA levels of this anti-thrombotic mediator in both un-stimulated and *R. conorii*-stimulated cells suggesting anti-thrombotic net effects of silencing DKK-1 in endothelial cells (Figure 6). In fact, silencing of DKK-1 resulted in a significant decrease in TF in *R. conorii*-exposed cells even compared with un-stimulated non-silenced cells (Figure 6). As for PAI-1 mRNA levels, there were no significant differences between silenced and non-silenced HUVECs in either un-stimulated or *R. conorii*-exposed cells (Fig. 6).

### Recombinant DKK-1 attenuates, but does not abolish, the suppressive effect of silencing DKK-1 on IL-6 release in HUVECs

**Figure 7:**
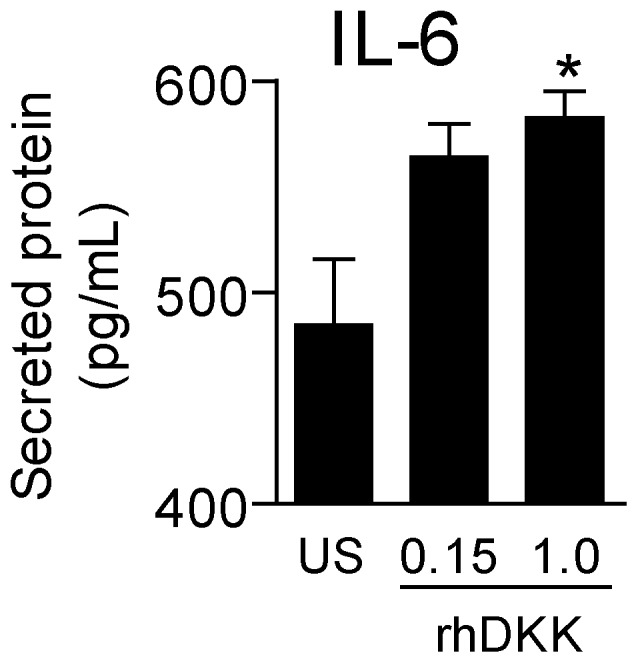
The effect of different concentrations of recombinant DKK-1 (µg/ml) on the release of IL-6 in HUVECs in the presence of siRNA probes to silence DKK-1 (culture time 24 hours). Data are given as mean±SEM (n = 4). *P<.05 vs. un-stimulated cells (US).

To further explore the role of DKK-1 in endothelial-related inflammation, we examined if recombinant DKK-1 could counteract the down-regulatory effect of silencing DKK-1 on IL-6 release. As shown in Figure 7, when adding recombinant DKK-1 to HUVECs that had silenced DKK-1, there was a dose-dependent increase in IL-6 release reaching statistical significance at a concentration of 1 µg/ml. However, although significant, the increase was rather modest (20%), and the addition of recombinant DKK-1 did not reverse the inhibitory effect of siDKK-1 on IL-6 release. This may suggest that it is the intracellular and not the secreted form of DKK-1 that is of most importance for the effect of DKK-1 on inflammatory mediators in endothelial cells.

## Discussion

The infection of endothelial cells and subsequent perivascular infiltration of leukocyte subpopulations is a major feature of infection with *R. conorii* and other SFG rickettsioses [2], [4]. This inflammatory interaction between *R. conorii* and endothelial cells involves the release of inflammatory cytokines and chemokines as well as up-regulation of adhesion molecules on endothelial cells and leukocytes [2], [4], [14]. The inflammatory response within the endothelium may be of importance for clearance of the bacteriae, whereas an overwhelming and persistent inflammatory response could also contribute to tissue damage and disease progression. In the present study we report for the first time an interaction between *R. conorii* and the Wnt pathway in the endothelium, with a decreased release of endothelial-derived DKK-1 in *R. conorii* exposed cells. Based on our finding in siDKK-1 transfected cells, with markedly attenuated inflammatory responses in cells with decreased DKK-1 expression, it is tempting to hypothesize that the *R. conorii*-induced down-regulation of endothelial-derived DKK-1 may reflect an immune evasion mechanism that facilitates rickettsial infection in the vascular bed.

Activation of the Wnt signaling pathway has been suggested to promote inflammation [7]–[10], [12], but there are also some reports indicating that this pathway could be involved in anti-inflammatory responses. Hence, recent studies suggest that DKK-1, an inhibitor of the Wnt signaling pathway, possess inflammatory properties. DKK-1 has been shown to trigger inflammation-induced bone loss [6], [21]. In endothelial cells, DKK-1 has been found to promote angiogenesis and enhance the inflammatory interaction between platelets and endothelial cells [11], [22]. In the present study we extend these findings by showing that silencing DKK-1 markedly attenuated the inflammatory response to heat-inactivated *R. conorii* in HUVECs with down-regulatory effects on IL-6, GROα and IL-8 at both mRNA and protein level. Moreover, we show that the effect of silencing DKK-1 in HUVECs is not restricted to inflammation. Down-regulation of DKK-1 in *R. conorii*-exposed HUVECs attenuated TF expression and enhanced thrombomodulin expression, suggesting pro-thrombotic net effect of DKK-1. Our findings further support a role for DKK-1 in vascular inflammation and atherothrombosis, and neutralization of DKK-1 could potentially represent a therapeutic target in relevant disorders.

Inflammatory stimuli such as TNFα have been shown to induce enhanced DKK-1 release in various cells [6]. Patients with MSF have previously been reported to have an early rise in TNFα and other inflammatory mediators [23]. It is therefore noteworthy that we found that patients with *R. conorii* infection had DKK-1 levels within the range of healthy controls when attending the hospital and before any specific treatment. However, endothelial cells release large amounts of DKK-1 upon activation, and the capacity of *R. conorii* to down-regulate DKK-1 in these cells, as opposed to its enhancing effect on IL-6 and IL-8, as shown in the present study, could counteract the increase in DKK-1 in relation to inflammatory stimuli in MSF patients. Yet, although heat-inactivated *R. conorii* down-regulated the release of DKK-1 from endothelial cells, there was no initial decrease in DKK-1 levels in serum in patients with *R. conorii* infection. This could potentially reflect contribution of other cells than endothelial cells to DKK-1 levels in serum. In fact, while *R. conorii* decreased the release of DKK-1 in HUVECs, it enhanced the release of DKK-1 in platelets and whole blood culture. In contrast to serum levels of DKK-1 at baseline, there was a significant increase in DKK-1 levels after 7 days. The reason for this pattern is at present unclear. Based on the ability of *R. conorii* to attenuate DKK-1 release, the possible clearance of *R. conorii* at time point 2 could contribute to a late increase in DKK-1. Second, the late increase could also be secondary to effects of inflammatory cytokines released during *R. conorii* infection known to induce DKK-1 release (e.g. TNFα). Nonetheless, our findings suggest that *R. conorii* affects DKK-1 and inflammatory cytokines differently both *in vivo* and *in vitro* in endothelial cells.

Immune evasion is of importance for the survival of microbes within the host, and such mechanisms also seem to be related to Rickettsial infection involving selection of inteferon-γ resistant strains, evasion of phagosomes and induction of anti-apoptotic mechanisms in endothelial cells [2], [4]. The production of inflammatory cytokines such as IL-6, IL-8, IL-12 and chemokines is crucial in the innate and adaptive immune responses to infections, and some bacterial pathogens have evolved mechanisms for attenuating cytokine production by host cells, which modifies the host's subsequent immune response [3]. Our findings in the present study could suggest that such mechanisms might be involved in immune evasion of *R. conorii* through its ability to down-regulate DKK-1 in endothelial cells. The Wnt signaling pathway has been linked to immune evasion mechanisms in relation to malignancies [24], and interestingly, recent studies indicate that Wnt signaling could be implicated in immune evasion in Mycobacteria and salmonella infection through anti-inflammatory and anti-apoptotic mechanisms, respectively. [25], [26] Our findings herein may suggest that the Wnt signaling pathway could also be involved in *R. conorii* related immune evasion by its ability to down-regulate DKK-1 expression in endothelial cells. The anti-apoptotic effects of DKK-1 may further support such a notion. [27], [28]. The current study has some limitations such as the use of heat-inactivated as opposed to live bacteria and a relative low number of patients with MSF. However, although our data are preliminary, we suggest that the capacity of *R. conorii* to down-regulate endothelial-derived DKK-1 as well as the ability of silencing DKK-1 to attenuate *R. conorii*-induced inflammatory responses in endothelial cells could reflect a novel mechanism by which *R. conorii* escapes the immune response at the site of infection. Yet, further studies are needed to establish this hypothesis as an important mechanism in SFG rickettsioses. Such studies should comprise more mechanistic studies including intervention studies in mice models for *R. conorii* infection.
